# Physical Health Monitoring of Children and Young Person Prescribed Antipsychotic Medications in Child and Family Health Devon

**DOI:** 10.1192/bjo.2026.11817

**Published:** 2026-06-30

**Authors:** Victor Osundeyi Osundeyi, Oluyemi Babalola, Reju Budhathoki

**Affiliations:** Child & Family Health Devon, Exeter, United Kingdom

## Abstract

**Aims:**

The aim of this clinical audit was to evaluate adherence to national guidelines and standards for antipsychotic prescription monitoring in children and young person attending CAMHS Devon, identifying areas for improvement and ensure that they receive safe and effective treatment. It was a re-audit of clinical audits that was done between June 2019 and February 2020 [3]. We aim to provide the various clinical indications and diagnoses for prescribing the antipsychotics, as well as to evaluate the quality of care and the standard of monitoring of antipsychotic prescribing. Our objective was to improve the quality of care and physical health monitoring of children and young person who are prescribed antipsychotic medications. This will contribute to ensure that children and young person receiving antipsychotic medications receive safe, high quality and effective care

**Methods:**

This audit was registered with DPT NHS trust clinical improvement team in September 2024 after attending a drop-in session with them for guidance and necessary support. Our evidence-based standards were POMH-UK audit of 2010 and 2012, CAMHS audit, 2019 and NICE guidelines. This was a retrospective re-audit looking at the records of all children and adolescents under the care of the entire Devon CAMHS teams currently prescribed antipsychotic medications.

We screened the records of all the caseloads [N=743 cases] that were opened to the 24 clinicians/prescribers working across the 3 CAMHS teams in CFHD, looking through their clinical notes on the electronic records [SystmOne and archives of carenotes]. We subsequently selected cases [n=74] of children and adolescents who met our inclusion criteria of been prescribed antipsychotic medications within the period of interest, ***July 2023 to August 2024***. The data collected were similar [with slight modification] to the previous audits and this was compiled and analysed using Microsoft excel. The data was collected in this way in order to make it easy for comparisons.

- Demographic details.
- Psychiatric diagnoses.
- Information about other clinical indications.
- Information about antipsychotics and other medications currently prescribed.
- Information about physical health screening and side effect monitoring.
- Information about medication review.

Comparisons were also made between the data from this audit and the previous ones POMH 2010, 2012 and Devon Audit, 2019.

**Results:**

We found a good standard of practice in terms of adequate documentation of the clinical indications and reasons for prescribing antipsychotic medications for about 10% [n-74] of children and young person attending our service. Regarding mental health diagnoses, off licence prescriptions and targeting specific symptoms, 65% [n-48] of cases prescribed antipsychotics had confirmed diagnosis of ASD and about 10% had ICD 10 diagnosis F20-29. About 59% of the prescriptions and oversight were done and under the supervision of consultant Child and Adolescent Psychiatrists. Aripiprazole was the most prescribed antipsychotic in 35% of cases and Melatonin in 46% of other medications prescribed. Evidence of physical health screenings were documented in almost 91% of those on antipsychotic medications. The most common side effects reported were weight gain [12%], tiredness [7%] and elevated Prolactin level in [3%]. There was no documented evidence of formal assessment of extrapyramidal side effect in all the cases and in 9% of cases there was no evidence of at least one physical health checks within the period of audit. All the identified 74 cases had medication review and there was no use of typical antipsychotic or polypharmacy prescription, which would be considered as a safe prescribing practice.

**Conclusion:**

The selection of antipsychotics for children and adolescents should include an evaluation of their individual therapeutic benefits, safety profiles and an approval status as per national formulary for use in this population.

